# GM-CSF promotes a supportive adipose and lung microenvironment in metastatic breast cancer

**DOI:** 10.18632/oncoscience.371

**Published:** 2017-10-23

**Authors:** Francesca Reggiani, Francesco Bertolini

**Affiliations:** Laboratory of Hematology-Oncology, European Institute of Oncology, Milan, Italy

**Keywords:** GM-CSF, Adipose tissue, breast cancer, neutrophils

The pro-tumorigenic role of progenitor cells resident in the white adipose tissue (WAT) has been demonstrated in preclinical models of breast cancer, where these cells had a significant impact on tumor angiogenesis, local tumor growth, and lung metastasis [1, 2]. Two distinct sub- populations of WAT-derived progenitors were identified, adipose mesenchymal progenitors (ASCs) and endothelial progenitors (EPCs), both of which contributed to tumor development in xenograft models [2]. In case of obesity, the number of these adipose progenitors is increased [1], so that they can play a relevant role in breast cancer patients with concomitant obesity or overweight.

We recently demonstrated the existence of a regulatory axis between breast cancer cells and WAT- derived progenitors [3]. Granulocyte macrophage colony- stimulating factor (GM-CSF) was suggested as the tumor-released factor, which induced the expression of GM-CSF itself and metallopeptidase 9 (MMP9) in adipose progenitors. These findings were collected *in vitro* and in xenograft models, using different triple negative breast cancer lines and primary adipose progenitors. In parallel with other studies, which also reported an increased expression of the two molecules in the context of breast cancer [4, 5], we also described that the higher release was due to transcription up-regulation occurring in WAT- derived progenitors, when exposed to tumor cells [3]. Both ASCs and EPCs produced higher levels of GM-CSF and MMP9 in presence of breast cancer, further supporting the cooperation in tumor malignancy [3].

The ablation of GM-CSF in tumor cells, achieved through siRNA delivery, prevented GM-CSF and MMP9 up-regulation in WAT-derived progenitors [3]. This suggested that GM-CSF may be required to alter WAT progenitors expression and to shift the adipose stroma to a supportive tumor microenvironment [3]. GM-CSF knock- down in xenografts or its neutralization in syngeneic diet-induced obese models was associated to a significant reduction of primary tumor growth, lung metastasis, and neoplastic angiogenesis and an increase in the number of immunosuppressive cells [3]. This is in contrast to some papers reporting an anti-tumor effect of GM-CSF in the context of breast cancer [6].

Another very recent paper identified GM-CSF as a crucial factor in the establishment of the pre-metastatic niche in lungs, collected from breast tumor-bearing mice [7]. Plasmatic levels of GM-CSF were significantly increased in mice with higher adiposity and directly correlated to tumor growth [7]. The increase of GM- CSF release in response to breast cancer progression was significant in obese mice, but not in lean mice, and more evident at the later stages of tumor progression [7]. Higher levels of plasma GM-CSF were also detected in our xenograft models based upon co-injection breast cancer cells and WAT progenitors, compared to mice injected with tumor cells alone [3]. These data support the role of adipose progenitors as the primary source of GM-CSF released into circulation. The effect was found to be exacerbated in obesity [7], possibly due to the increased number of WAT progenitors [1]. The GM- CSF-enriched serum promoted the differentiation of bone marrow-derived cells into neutrophils with tumor- associated phenotype (TANs), found to be accumulated in lungs of these mice [7]. Consequently, the generation of a pre-metastatic niche in WAT-poor organs might be a direct consequence of the imbalanced secretion of GM-CSF in the adipose tissue surrounding the primary neoplastic lesion. GM-CSF alteration directly correlates to the establishment of systemic inflammation which affects also WAT-poor organs, such as lungs.

**Figure 1:**
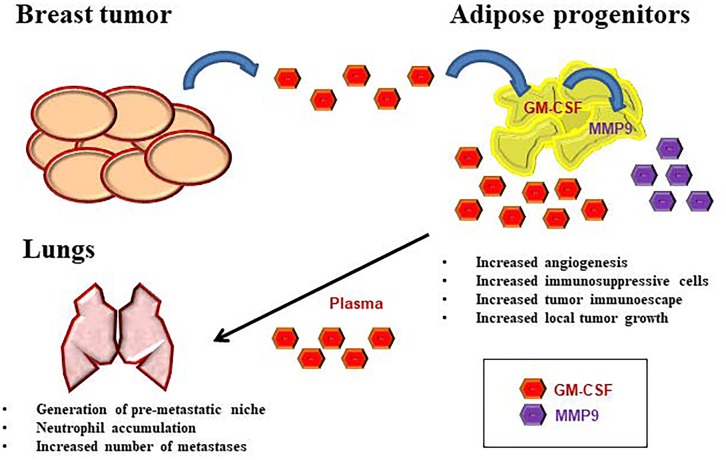
The role of GM-CSF in metastatic breast cancer Tumor cells express high levels of GM-CSF in the neoplastic microenvironment which in turn increases GM-CSF and MMP9 expression in WAT-derived progenitors in the surrounding adipose tissue. GM-CSF release into circulation increases lung metastasis, thus promoting the differentiation of tumor-associated neutrophils (TANs) and their accumulation in the lung pre-metastatic niche.

The mechanism by which GM-CSF acts on different cell types in tumor microenvironment might be dose- and cell-dependent. When the factor is present at relatively low levels, its anti-tumor effects may be due to the activation of dendritic cells, as in case of tumor cell vaccine [8]. In contrast, when GM-CSF release is increased in presence of obesity and primary tumor, its activity is mainly immunosuppressive, enriching adipose microenvironment of myeloid-derived suppressor cells (MDSCs), tumor- associated macrophages (TAMs), T-regulatory (T-reg) cells [3] and circulating/lung TANs [7]. In addition, GM-CSF supports angiogenesis in primary breast tumor and the release of metalloproteases, further triggering cancer invasion and metastatic spread [3]. The schematic representation of the axis existing between breast tumor, WAT and lungs is reported in Figure 1.

Further investigations should be addressed to clarify the exact molecular signature associated with GM-CSF over-expression in adipose progenitors and its relevance in other neoplastic diseases.
